# An elderly patient with depression and a suicide attempt during the COVID-19 pandemic: a case report

**DOI:** 10.3389/fpsyt.2023.1151482

**Published:** 2023-09-28

**Authors:** Hajar Mohd Salleh Sahimi, Marhani Midin, Jane Tze Yn Lim, Mohd Wafiy Ariffin Anwar, Farah Deena Abdul Samad, Nurul Ain Mohamad Kamal

**Affiliations:** ^1^Department of Psychiatry, Faculty of Medicine, Universiti Kebangsaan Malaysia, Cheras, Malaysia; ^2^Hospital Canselor Tuanku Muhriz, Bandar Tun Razak, Kuala Lumpur, Malaysia

**Keywords:** geriatric depression, COVID-19, lockdown, pesticide poisoning, geriatric suicide

## Abstract

**Objective:**

Elderly individuals are among the age groups with the highest risk of suicide. The coronavirus (COVID-19) pandemic forced isolation and resulted in an increased risk of depression, hopelessness, and perceived burdensomeness among the elderly, thereby increasing the risk of suicide.

**Methods:**

This is a case report of an elderly single retired school principal with obsessive-compulsive personality traits who developed depression with psychotic symptoms after being isolated following the movement control order (MCO) during the COVID-19 pandemic. The social isolation led to feelings of loneliness and hopelessness. The patient’s depressive symptoms worsened after he developed physical illnesses, such as eye floaters, that affected his daily activities. This caused him to have suicidal ideation to the extent that he attempted suicide by ingesting 90 mL of pesticide. Two weeks prior to the attempt, he updated his will and asked his friend to keep it. After the suicide attempt, he vomited and had diarrhea and epigastric pain. He called his friend, who brought him to the hospital emergency room (ER). He was resuscitated and subsequently admitted to the intensive care unit (ICU). After being medically stabilized, he was transferred to the psychiatric ward, where further treatment was administered for his depression. His depressive symptoms and suicidal ideation improved after he was administered antidepressants and psychotherapy.

**Results:**

The impact of the COVID pandemic has led to a surge in mental health issues such as anxiety and depression. The elderly are among the highest-risk groups of individuals to contract or die of COVID-19 infection, and they are also the most likely to develop mental health issues related to the pandemic. Furthermore, the risk of death by suicide is highest in this age group due to physical illness, social isolation, and the lack of a support system. This case also highlights the need for awareness of suicidal ideation screening among non-medical healthcare professionals and religious organizations to avoid the treatment gap.

**Conclusion:**

It is essential to enhance suicide risk assessment and management among the elderly after the COVID-19 pandemic.

## Introduction

COVID-19 was declared a pandemic by the World Health Organization (WHO) on 11th March 2020. The elderly population is at a higher risk of mortality (1), with 80% of deaths in the United States (US) among adults aged 65 years and over (2), more than 90% of deaths in Europe (3), and 80% of deaths in China among adults aged over 60 (4). The COVID-19 pandemic has led to a surge in mental health issues, with high rates of anxiety and depression at 25.6 and 23.1%, respectively, observed in an international collaborative study conducted during the first quarter of 2020 (5). During the third wave of the pandemic in Malaysia, 70% of the elderly experienced moderate to severe depression, and 43.5% had mild to severe anxiety (6). Suicide rates also increased during the pandemic (7, 8). Compared to other age groups, the elderly population was at a significantly higher risk for completed suicide (9).

Suicide is a complex, dynamic, and multifactorial behavior. Studies on the association of infectious epidemics with suicide, self-harm, and thoughts of suicide or self-harm found an increase in suicides among the elderly in Hong Kong during the SARS pandemic (10). Elderly individuals in the age group of 70–79 years have been identified as having the highest risk of suicide (11) among all age groups. Due to this grave concern regarding suicide among the elderly population, studies have looked into possible factors mediating suicidal behavior, which seem to involve the interaction of biological or psychological diseases (11).

Among the risk factors for developing elderly depression is the presence of a pre-existing physical illness. During the COVID-19 pandemic, elderly individuals were significantly affected by the enforcement of lockdown control. Those with pre-existing physical or mental illnesses might not have had the opportunity to receive early medical help due to resource rationing (12), which might have led to a sense of “being a burden” on society (13). Physical health problems were recorded as a suicide precipitant for 50% of the older decedents (14).

Due to multiple medical comorbidities, the elderly were among those with the highest risk of mortality due to COVID-19 (15). This led to another risk factor for elderly depression, which is the presence of comorbid mental illnesses such as anxiety or personality traits. A heightened fear of contracting COVID-19, especially among the elderly, was observed (16). Anxiety during COVID-19 was shown to be positively correlated with hopelessness (17). Data collected during a curfew in Turkey showed that the anxiety levels of the elderly were negatively affected during the pandemic and might have led to depression (18).

Loneliness caused by lockdown restrictions was identified as another risk factor contributing to elderly depression, leading to self-harm and suicide due to a sense of isolation, a lack of or reduced face-to-face contact with their families and social networks, a disruption in the normal routine, and feelings of entrapment (19). There has been evidence of a high level of loneliness during the COVID-19 pandemic (20), recognized as a major public health concern, especially among the elderly. Social isolation often results in loneliness, a factor significantly associated with depression in the elderly (21).

Depression in the elderly is a significant factor associated with suicide. A cross-sectional study in Indonesia during the COVID-19 pandemic revealed that 53.6% of 457 elderly individuals experienced depression, with a higher risk among those who were fully dependent (22). Furthermore, the deterioration of depressive symptoms was evidenced in older adults aged more than 50 years during the first wave of COVID-19 in Europe. Approximately 40% of women and 31% of men classified as extremely depressed 5 years before the pandemic were reported to have more depressive symptoms later in 2020, whereas 10% of those at a very low risk of depression reported earlier had increased depressive issues during the pandemic (23). The vulnerability to depression causes a negative inferential style that perceives any adverse life events as hopeless, which may lead to suicidal ideations and behaviors (24). Individuals with negative cognitive styles tend to infer flawed and underserving characteristics about themselves (25) and to generalize that negative events (such as the COVID-19 pandemic) will lead to many other negative consequences (25).

This case report highlights a case of elderly depression, predisposed by risk factors such as pre-existing physical and mental illnesses, personality traits, and loneliness due to the COVID-19 pandemic, which eventually led to a serious suicide attempt.

## Case report

Mr. L., a 64-year-old Chinese gentleman who is a single retired school principal, was brought to the emergency department by his friend for trying to take his own life. He had ingested 90 mL of pesticide (containing chlorpyrifos), resulting in severe gastrointestinal side effects. Mr. L. had consumed the pesticide all at once. He was alone at home at the time. He had not planned ahead. His thought process at the time of ingestion was his anxiety about the severity of his depression and his high antidepressant dosage, in addition to his prognosis. He had bought the pesticide more than a year earlier due to an ant infestation issue at his home and had kept it on a table below his medicine cabinet. Immediately after ingesting the pesticide, he began to feel nauseous. He had diarrhea, which caused him to run to the toilet, where he accidentally fell over a bucket and sustained a superficial laceration wound on his right neck. He then began calling his friends and brother to take him to the hospital due to the discomfort. He had a will prepared many years ago but did not bid farewell. Two weeks before he attempted to take his own life; he informed his friend where he kept his will. Upon reaching the hospital, he developed respiratory distress, requiring ventilatory support, which was complicated by hospital-acquired pneumonia. His medical stabilization took nearly 4 weeks before he was transferred to the psychiatric ward.

Mr. L.’s depressive episode started in early 2021, brought about by the changes during the COVID-19 pandemic and the lockdown restrictions imposed during the Malaysia Movement Control Order (MCO), equivalent to the national “lockdown.” He experienced low mood and a loss of interest and appetite, in addition to significantly compromised sleep, concentration, and energy levels. His friends also noticed that he had persecutory delusions, in which he believed that his neighbors were talking negatively about him. Moreover, he experienced visual hallucinations in which he could see devils or children wandering up and down the sidewalks when out with friends. He denied symptoms that suggested mania, hypomania, or anxiety. During the MCO, he was forced to live a solitary lifestyle when his yearly social trips to China had to be canceled. He was also cut off from the social circle he used to meet with daily for morning exercises and meals before the MCO. His social isolation and loneliness were further compounded by his visual impairment when he experienced floaters in both eyes, along with the fear of permanent blindness and complications from surgery. As a voracious reader and writer, he found life too burdensome under these circumstances, which further exacerbated his depressive symptoms. His church friends had tried multiple times to convince him to get himself admitted to a psychiatric ward. However, it was only after 1 and a half years of experiencing depressive symptoms that he finally agreed to visit a private psychiatrist. The psychiatrist started him on escitalopram and conducted psychotherapy sessions with him. Nevertheless, he experienced only a slight amelioration of these symptoms. The worsening of his untreated depressive symptoms over time eventually led to feelings of hopelessness, worthlessness, and suicidality as his future was perceived to be bleak. He also experienced mood-congruent psychotic symptoms.

Mr. L. had obsessive-compulsive personality traits that made him averse to change. His friends and family had always described him as a meticulous and clean individual. His home was neat and well-organized; however, he would frequently express his disappointment that his house was not orderly and clean enough. For instance, he would not keep any fresh food in his residence because he feared it would attract flies. He refused to buy a washing machine, a microwave oven, or a refrigerator because he felt they would not clean well enough and would take up room, and he felt that that would be another thing he had to clean. He used to wash all his clothes and bed linen by hand. However, as he became more depressed, he found it challenging to keep up. Despite having some symptoms of obsessive-compulsive personality traits, he did not fulfill the criteria for obsessive-compulsive personality disorder (OCPD) based on the Diagnostic and Statistical Manual of Mental Disorders (DSM-5) (26), and he did not have a psychometric test done to confirm a personality disorder diagnosis.

Mr. L. began experiencing floaters in both eyes during the MCO. He had sought two consultations with an ophthalmologist but waited for his condition to worsen before deciding to undergo surgery out of fear of complications and permanent blindness.

Mr. L. is a retired headmaster who premorbidly had an active social life, where he would travel overseas with his friends and would be heavily involved with a local church organization. His good friend, who advised him to seek psychiatric help and was a good social support, was also among the church organization members.

The provisional diagnosis according to DSM-5 (26) was “major depressive disorder with mood incongruent psychotic features,” as he fulfilled more than five symptoms from criteria A that lasted more than 2 weeks (low mood, anhedonia, poor appetite and sleep, poor concentration and energy levels, and suicidal ideations). He also met criteria B, in which his symptoms were severe enough to impair his social functioning – these were not due to any substance or medical illness (criteria C). A differential diagnosis for Mr. L.’s condition according to DSM-5 (26) was “adjustment disorder with depressed mood.” He fulfilled criteria A in that his symptoms were in response to an identifiable stressor (COVID-19 lockdown isolation) within 3 months of the stressor. He also met criteria B in that he developed marked distress and functional impairment. However, his symptoms were better explained by a diagnosis of “major depressive disorder”; hence, criterion C was not satisfied. In the ward, he was started on admission on an antidepressant (sertraline 100 mg ON) and an antipsychotic (olanzapine 5 mg ON). After discharge, he attended the outpatient clinic four times for follow-up; his mood significantly improved, and his psychotic symptoms resolved. Olanzapine was stopped, and he maintained well on sertraline 50 mg ON. He was offered a referral to psychotherapy, but he declined due to logistical reasons.

## Discussion

Elderly suicide is a complex clinical and public health concern. The changes brought about by COVID-19 have made the situation even more challenging. Suicide prevention among older adults is difficult (27). Primary healthcare providers who regularly treat elderly patients are the main gatekeepers for suicide prevention (28); they must inquire about mental illness and any evidence of suicidal ideation, bearing in mind that elderly patients may present with physical symptoms as opposed to classic depressive symptoms, which makes detection quite challenging (29). In Mr. L.’s case, he had no contact with primary healthcare providers. Nevertheless, he had attended his ophthalmology appointment a couple of times, and retrospectively, these contacts with healthcare professionals occurred at a critical period of his deteriorating depressive symptoms. Only after 1 year and a half of untreated depression did his friend suggest that he see a psychiatrist.

Screening for suicidal ideation should, therefore, be opportunistic in high-risk subgroups. These subgroups include individuals with depressive illnesses, those with a previous history of suicide attempts, those with comorbid physical illnesses, and those who are socially isolated (27). The high index of suspicion for opportunistic screening, for example, in Mr. L.’s case, might have yielded early detection of depressive episodes and the initiation of treatment. Unfortunately, the ophthalmologist did not detect his depressive symptoms, which led to a late diagnosis and initiation of treatment. One study showed that a patient’s age, gender, race, and comorbid medical conditions affect a medical practitioner’s awareness of mental health problems. Other barriers include the segregation of knowledge when medical fields are too subspecialized, causing a lack of mental health knowledge and skills among non-mental health medical specialties (30).

It took Mr. L. 1 and a half years to seek help from a mental health provider. An element of self-stigma and poor insight/awareness might have contributed to this. Self-stigma has been shown to exist among elderly individuals with depression (31). For example, one study showed that Korean and Russian elderly individuals endorsed views of having depression as “being weak” (32). Other than intra-personal stigma, the presence of “inter-personal stigma” (public prejudice and discrimination toward people with mental illness) and “structural stigma” (poor legislation and policies on mental illness or inadequate mental health services) could also be the reason for the late detection of mental illness. Among suggestions for improving stigma are developing policies that are inclusive of mental illness, promoting mental health to improve public mental health literacy, task-shifting and sharing with the community for psychosocial support, and improving access to mental health care (33).

Mr. L. had good social support from his church organization. Religious organizations could be another avenue for early detection of mental health issues, and with further training, professional counseling sessions can also be conducted by these organizations. One study in the United Kingdom showed that Christian clergy frequently encountered mental health cases, and approximately 60–80% were regularly referred to a healthcare professional (34). A collaboration among religious organizations and mental health providers may reduce the burden on tertiary psychiatric centers, allowing individuals to receive help earlier (34–36). More training on the early detection of mental health issues among the elderly population can be conducted to increase awareness of the roles of each provider. This training should include the recognition of certain red flag phrases with hidden messages, such as a perception that the future is persistently negative and hopeless, such as “I have nothing to live for,” extreme guilt, such as “I am a burden,” a sense of entrapment, such as “there is nothing else I can do,” or a sense of shame, such as “I cannot continue tainting the family name” (37). Trained providers who are mindful of such challenges can help improve the detection of those at risk for suicide (38), and with optimum treatment, suicide risk can be mitigated (34).

With the rise of the unbidden COVID-19 disease since November 2019, the WHO has recommended mandatory self-isolation for older adults due to their vulnerability to infections. Strict movement control orders (MCOs), physical distancing, and the sense of being disconnected are among the factors that have been shown to contribute to increased depression and anxiety (39). These factors were reflected in the case of Mr. L., a former school principal who had always been independent. He was actively involved in his church and had a close circle of friends. MCO had sadly robbed him of these, resulting in the emergence of depressive symptoms, which later led him to attempt suicide. To combat isolated elderly individuals, a comprehensive evaluation, followed by a delineation of the roles and responsibilities of various institutions, starting from the family unit to healthcare professionals, and the development of an individual strategy, have been proposed (40). Individual strategies include utilizing technological opportunities for peer communication, social support, and environment, using cognitive behavioral therapies, and other culturally appropriate approaches.

An age-friendly student–senior pilot program was conducted to combat loneliness and isolation among the elderly through 30–60 min of phone calls with older adults two to five times a week for 6 weeks during the COVID-19 pandemic. It showed statistically significant changes and reported benefits and outcomes (41). Interventions at the population level should particularly focus on improving social contact and support, especially considering that low social contact is one of the attributable risk factors (42). Fischer et al. (43) discovered a significant reduction in completed suicide among elderly people who utilized telephone helplines.

Mr. L.’s depressive episode worsened when he anticipated the loss of visual ability and imagined the worst possible outcome as blindness. On top of being robbed of his physical independence during the MCO, his quality of life and daily functioning were affected even further due to his worsening vision. Consequently, this became a predisposing factor to his suicide attempt.

Optimizing physical health is of paramount importance in addressing the risk of depression-related suicide, secondary to the loss of physical health. A retrospective study in Italy found that 63.41% of elderly men took their own lives due to physical illness, using suicide to ease their physical suffering (11). Half of the elderly participants who attempted suicide due to physical illness were reported to be twice as likely to be depressed (44). A systematic review also supported this finding, revealing a significant association between functional disability and suicidal behavior. Moreover, the study observed that depression is a mediator between these associations (45).

The suicide methods employed by the elderly are much more lethal compared to those employed by younger age groups. They are also less likely to survive as they are fragile. In Italy, the most common method was hanging, followed by falling from a height, drowning, and firearms. Pesticides, knives, or asphyxiation are less common methods (11). Pesticide ingestion was also a popular method for suicide in South Korea. This method was found to be employed more among men and the elderly, and it was more common in rural than in non-rural areas (46). In Taiwan, pesticides, herbicides, and psychiatric drugs were the most common methods employed for suicide (47). In Malaysia, hanging was the most common method for suicide among the elderly, accounting for 56.5% of cases recorded in the National Suicide Registry Malaysia (NSRM) in 2009, followed by jumping from a height (13.1%) and exposure to unspecified chemicals (13.1%) (48). In Mr. L.’s case, he ingested 90 mL of pesticide (containing chlorpyrifos), which was readily available at home for pest control.

A strategic intervention in suicide prevention includes reducing access to lethal means of suicide. The usage of pesticides as a method for suicide in South Korea rapidly declined after paraquat was banned in 2012 (46). In 2018, Taiwan also imposed a ban on paraquat. It was found that the suicide rate due to pesticides was reduced by 37% in the next year. The rates were reduced most among those aged 65 years and older, men, and those living in rural areas (49). In Malaysia, paraquat was banned from 2004 to 2006. However, the ban was lifted a year later; the National Poison Centre reported increased paraquat poisoning after the ban was lifted, with a majority of cases reportedly due to suicide attempts. Malaysia has re-implemented the ban on paraquat since January 2020 (50). Preventing suicide through pesticide regulation is also an available approach that can be taken by the government and stakeholders (51).

Mr. L.’s condition improved after he was transferred to the psychiatric ward and after his medication was optimized; however, the treating team remained cognizant of the risk of him attempting suicide again. Studies have shown that the risk of re-attempting suicide is higher among those with cluster B personality disorder and alcohol use disorder, and in those who are older (52). Other than that, the greater lethality of the first attempt, the severity of depression, and the absence of a stable relationship may also contribute to a second suicide attempt (53). This highlights the importance of continuous suicide risk assessment and management with a collaborative approach involving the patient’s support system, from community organizations to friends. Furthermore, successful aging can be promoted by improved physical and mental health well-being, regular exercise, and lifestyle modification, thus decreasing suicidal ideation (42).

## Conclusion

Suicide in the elderly population is a multifaceted phenomenon. A comprehensive screening, prevention, and management plan should focus more on the elderly in view of the innately increased risk of suicide in this population. Clearly, in the midst of a pandemic such as COVID-19, elderly people with added risk factors, such as those with underlying physical illnesses and those who are socially isolated, should be vigorously screened and aggressively treated for depression and suicidal ideation.

## Data availability statement

The raw data supporting the conclusions of this article will be made available by the authors, without undue reservation.

## Ethics statement

Written informed consent was obtained from the individual(s) for the publication of any potentially identifiable images or data included in this article.

## Author contributions

HM and JL contributed to the conception and design of the study and wrote the first draft of the manuscript. MA organized the database. HM, MM, JL, MA, FA, and NM wrote sections of the manuscript. All authors contributed to the article and approved the submitted version.
